# Independent glial subtypes delay development and extend healthy lifespan upon reduced insulin-PI3K signalling

**DOI:** 10.1186/s12915-020-00854-9

**Published:** 2020-09-14

**Authors:** Nathaniel S. Woodling, Arjunan Rajasingam, Lucy J. Minkley, Alberto Rizzo, Linda Partridge

**Affiliations:** 1 Institute of Healthy Ageing and Department of Genetics, Evolution and Environment, University College London, Darwin Building, Gower Street, London, WC1E 6BT UK; 2 Max Planck Institute for Biology of Ageing, Joseph-Stelzmann-Strasse 9b, 50931 Cologne, Germany

**Keywords:** Ageing, Astrocytes, Glia, Insulin signalling, Lifespan

## Abstract

**Background:**

The increasing age of global populations highlights the urgent need to understand the biological underpinnings of ageing. To this end, inhibition of the insulin/insulin-like signalling (IIS) pathway can extend healthy lifespan in diverse animal species, but with trade-offs including delayed development. It is possible that distinct cell types underlie effects on development and ageing; cell-type-specific strategies could therefore potentially avoid negative trade-offs when targeting diseases of ageing, including prevalent neurodegenerative diseases. The highly conserved diversity of neuronal and non-neuronal (glial) cell types in the *Drosophila* nervous system makes it an attractive system to address this possibility. We have thus investigated whether IIS in distinct glial cell populations differentially modulates development and lifespan in *Drosophila*.

**Results:**

We report here that glia-specific IIS inhibition, using several genetic means, delays development while extending healthy lifespan. The effects on lifespan can be recapitulated by adult-onset IIS inhibition, whereas developmental IIS inhibition is dispensable for modulation of lifespan. Notably, the effects we observe on both lifespan and development act through the PI3K branch of the IIS pathway and are dependent on the transcription factor FOXO. Finally, IIS inhibition in several glial subtypes can delay development without extending lifespan, whereas the same manipulations in astrocyte-like glia alone are sufficient to extend lifespan without altering developmental timing.

**Conclusions:**

These findings reveal a role for distinct glial subpopulations in the organism-wide modulation of development and lifespan, with IIS in astrocyte-like glia contributing to lifespan modulation but not to developmental timing. Our results enable a more complete picture of the cell-type-specific effects of the IIS network, a pathway whose evolutionary conservation in humans make it tractable for therapeutic interventions. Our findings therefore underscore the necessity for cell-type-specific strategies to optimise interventions for the diseases of ageing.

## Background

The rapid and projected increase in life expectancy across industrialised nations [1] will bring with it a daunting prevalence of age-related co-morbidities, underscoring the need to better understand the biological underpinnings of ageing. To this end, increasing evidence supports the antagonistic pleiotropy theory, which posits that organismal senescence derives from single genes evolutionarily favoured for their beneficial effects in early life despite their detrimental effects in late life. These opposing effects can manifest in different tissues or cell types: for example, in the hypothetical example imagined by Williams in his original outline of the theory, a single gene might mediate both beneficial bone calcification during development and detrimental artery calcification in older age [2]. Decades of research have since identified several more concrete examples of cell-type-specific antagonistic pleiotropy: tumour suppressor genes, for instance, are essential for inhibiting cell division and promoting apoptosis in pre-malignant cells, but their action in otherwise healthy cells can promote both cellular and organismal senescence [3]. Perhaps the most widely studied example of antagonistic pleiotropy in ageing is the insulin/insulin-like growth factor (IGF) signalling (IIS) pathway, although few studies have detailed whether individual cell types and subtypes are responsible for each of its opposing pleiotropic effects.

IIS is a highly evolutionarily conserved nutrient sensing pathway responsible for the coordination of nutrition with growth and metabolism across numerous tissues. While this pathway is necessary for appropriate development, its continued activity in adulthood is lifespan-limiting in diverse eukaryotic species [4, 5]. Since the original identification of *daf-16* (the *C. elegans* insulin receptor orthologue) as a gene whose reduced activity can extend healthy lifespan [6], numerous studies have dissected the molecular underpinnings behind the effects of IIS on both development and lifespan. This work has identified a number of lifespan-extending interventions in diverse model organisms, including genetic deletion of the insulin receptor or its substrates [7–10], inhibition of the effector kinases PI3K [11] or RAS [12], or overcoming the inactivation of the transcription factor FOXO by IIS [13, 14]. In humans, single-nucleotide polymorphisms in the *FOXO3A* gene are associated with longevity in large-scale genome-wide association studies [15, 16], implying that IIS plays an evolutionarily conserved role in human ageing as well. However, lifespan extension from IIS inhibition often comes with trade-offs of delayed development and reduced growth [17]. At the organismal level, these effects appear to be governed by at least partially distinct molecular mechanisms: for example, while FOXO is required for lifespan extension from reduced IIS in *Drosophila*, FOXO is not required for the delayed development, reduced body size, or lowered fecundity from the same interventions [11].

It is likely that the pleiotropic effects of IIS inhibition also derive from diverse effects in distinct cell types, although few studies have extensively defined these cell types for both detrimental and beneficial effects. At the level of lifespan extension, IIS inhibition can promote longevity even when restricted to *Drosophila* muscle tissue [18], gut enterocytes [19], or the fat body (the *Drosophila* analogue of liver and adipose tissues) [13, 14], with non-overlapping molecular pathways exerting pro-longevity effects downstream of reduced IIS in these tissues [20]. Notably, the nervous system is one tissue where the effects of reduced IIS on both development and adult lifespan have been assessed. In mice, partial inactivation of the IGF-1 receptor throughout all brain cell types delays growth and increases mean lifespan in mice [21]. However, the possibility remains that IIS in individual nervous system cell types is responsible for effects on development and adult lifespan. IIS in glia, the non-neuronal cells of the nervous system, plays a central role in *Drosophila* nervous system development, both in reactivating quiescent neuroblasts [22, 23] and in coordinating neuronal differentiation among photoreceptors and optic lobe lamina neurons [24]. In adult flies, glia also appear to be important sites for IIS activity, as single-cell transcriptomic data from adult fly brains show enrichment for expression of the *Insulin-like Receptor (InR)* gene in glia compared to all subclasses of neurons [25]. In addition, glia are the class of cells that change their transcriptional profile most dramatically with age across human brain regions [26] and in *Drosophila* brains [25, 27], suggesting the possibility that glial IIS may be involved in ageing as well as development across species.

We have therefore asked whether glial IIS modulates both development and lifespan through distinct glial subclasses in *Drosophila*. We find that pan-glial IIS inhibition extends healthy lifespan and delays development, but without any impact on brain size or fecundity. We further describe that the effects on developmental timing and adult lifespan can be separated temporally by life-stage-specific IIS inhibition in glia. Finally, we find that the effects of glial IIS can also be separated spatially among glial subtypes, with reduced IIS in astrocyte-like glia able to extend lifespan without any effect on developmental timing, whereas its inhibition in other glial subtypes delays development without extending lifespan. Taken together, these findings present a robust example of cell-type-specific antagonistic pleiotropy in which a single molecular pathway exerts its opposing effects through distinct cell types, providing another layer of complexity to the understanding of ageing.

## Results

### Glial *Pten* over-expression extends lifespan and delays development

**Fig. 1:**
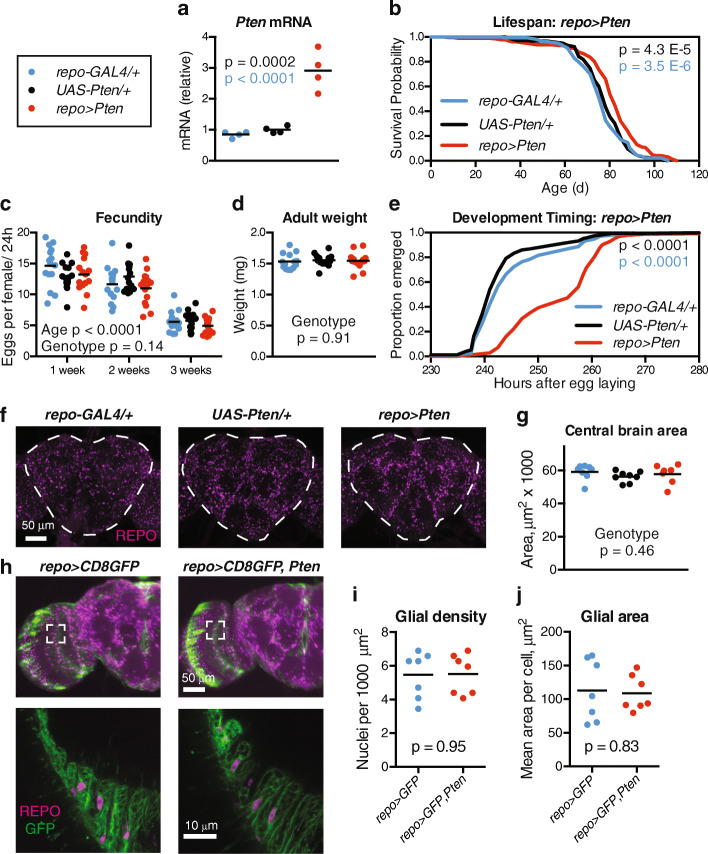
Glial *Pten* over-expression extends lifespan and delays development. **a** qPCR from head RNA shows over-expression of *Pten* in 1-week-old *w*^*Dah*^*;UAS-Pten/+;repo-GAL4/+* flies (red) compared to control *w*^*Dah*^*;+;repo-GAL4/+* (blue) and *w*^*Dah*^*;UAS-Pten/+;+* (black) flies. *n* = 4 replicates of 12 heads per replicate per genotype. **b** Survival curves show extended lifespan for *w*^*Dah*^*;UAS-Pten/+;repo-GAL4/+* flies (red) compared to control *w*^*Dah*^*;+;repo-GAL4/+* (blue) and *w*^*Dah*^*;UAS-Pten/+;+* (black) flies. *n* > 150 deaths counted per group. **c** The number of eggs laid by female flies of the indicated ages was not affected by genotype. *n* = 15 vials of 12 flies per vial per genotype. **d** The weight of 1-week-old female flies was not affected by genotype. *n* = 14 flies per genotype. **e** Egg-to-adult timing shows delayed development for *w*^*Dah*^*;UAS-Pten/+;repo-GAL4/+* flies (red) compared to control *w*^*Dah*^*;+;repo-GAL4/+* (blue) and *w*^*Dah*^*;UAS-Pten/+;+* (black) flies. *n* > 200 flies counted per group. **f** Confocal z-projections show glial nuclei immunostained for REPO (magenta) in the central brain. Dotted lines indicate traces of central brain areas. **g** Central brain areas were unchanged in 1-week-old *w*^*Dah*^*;UAS-Pten/+;repo-GAL4/+* flies (red) compared to control *w*^*Dah*^*;+;repo-GAL4/+* (blue) and *w*^*Dah*^*;UAS-Pten/+;+* (black) flies. *n* = 7–8 brains per group. **h** Confocal z-projections show glial nuclei immunostained for REPO (magenta) and glial membranes labelled with mCD8::GFP (green). Dotted lines indicate areas imaged at higher magnification below. **i**, **j** REPO-positive glial nuclei were counted and the extent of glia measured by GFP-positive area in the layer of glia shown in **h**. Both the density of glial nuclei and the extent of glial membranes were unaffected in 1-week-old *w*^*Dah*^*;UAS-Pten/+;repo-GAL4/UAS-mCD8::GFP* flies (red) compared to control *w*^*Dah*^*;+;repo-GAL4/UAS-mCD8::GFP* (blue) flies. *n* = 7 brains per group. *p* values are from Tukey’s multiple comparisons (**a**), log-rank test versus control group of that colour (**b**, **e**), 2-way ANOVA (**c**), 1-way ANOVA (**d**, **g**), or unpaired t-test (**i**, **j**)

To assess the effects of glial IIS on adult lifespan, we first tested over-expression of PTEN (Phosphatase and Tensin Homologue), a lipid phosphatase that antagonises the activity of PI3K and has previously been found to extend *Drosophila* lifespan when over-expressed in muscle [18] or pericerebral fat body [14]. Using the canonical glial driver *repo-GAL4*, we found that *Pten* over-expression in glia significantly increased lifespan (Fig. 1a, b, median + 5.9%, *p* = 4.3 × 10^−5^ versus the nearest control group). Flies with glial *Pten* over-expression showed an overall 36% reduced hazard of death (Cox hazard ratio 0.64, 95% confidence interval 0.52–0.80 compared to the nearest control group). Importantly, this lifespan extension was reproducible in independent experiments and with fly stocks backcrossed to either the outbred *w*^*Dah*^ or inbred *w*^*1118*^ strains (Additional file 1: Figure S1). We observed no change in fly weight or fecundity in these flies (Fig. 1c, d), but we found a significant ~ 16-h delay in developmental timing (Fig. 1e). Because PTEN over-expression can reduce cell size through cell-autonomous IIS inhibition in some tissues [28], we next assessed the overall nervous system size and glial cell size in our long-lived flies. However, we found no difference in adult brain size (Fig. 1f, g) or the estimated number and size of glial cells as defined by the extent of their GFP-labelled membranes (Fig. 1h–j). While seemingly at odds with previous findings that glial PI3K inhibition prevents neuroblast reactivation during development [22, 23], our data on the unaffected brain size in these flies suggest that larvae eventually bypass this delay and compensate with growth to full size, potentially through pathways that preserve nervous system growth in the absence of nutritional cues [29]. These findings suggest that glial IIS inhibition can extend lifespan at the cost of delayed development but without marked effects on adult size or fecundity.

### The lipid phosphatase activity of PTEN is essential for its lifespan-extending effects in glia

**Fig. 2:**
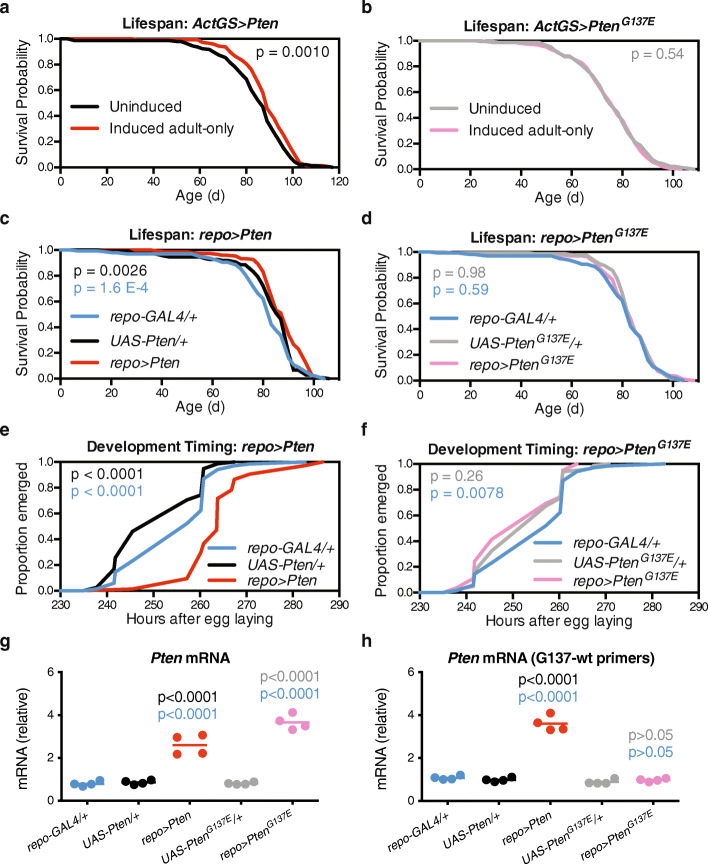
Over-expression of wild-type *Pten*, but not lipid phosphatase dead *Pten*, extends lifespan and delays development. **a**, **b** Survival curves show **a** extended lifespan for *w*^*Dah*^*;Actin-GeneSwitch/UAS-Pten;+* flies and **b** unchanged lifespan for *w*^*Dah*^*;Actin-GeneSwitch/UAS-Pten-G137E;+* flies reared on food containing 200 μM RU-486 (induced, red and pink curves) from 2 days of age compared to sibling flies reared on vehicle control food (uninduced, black and grey curves). **c**, **d** Survival curves show **c** extended lifespan for *w*^*Dah*^*;UAS-Pten/+;repo-GAL4/+* flies (red) compared to control *w*^*Dah*^*;+;repo-GAL4/+* (blue) and *w*^*Dah*^*;UAS-Pten/+;+* (black) flies, and **d** unchanged lifespan for *w*^*Dah*^*;UAS-Pten-G137E/+;repo-GAL4+* flies (pink) compared to control *w*^*Dah*^*;+;repo-GAL4/+* (blue) and *w*^*Dah*^*;UAS-Pten-G137E/+;+* (grey) flies. For all lifespans, *n* > 130 deaths counted per group. **e**, **f** Egg-to-adult timing shows **e** delayed development for *w*^*Dah*^*;UAS-Pten/+;repo-GAL4/+* flies (red) compared to control *w*^*Dah*^*;+;repo-GAL4/+* (blue) and *w*^*Dah*^*;UAS-Pten/+;+* (black) flies, and **f** no delay in development for *w*^*Dah*^*;UAS-Pten-G137E/+;repo-GAL4/+* flies (pink) compared to control *w*^*Dah*^*;+;repo-GAL4/+* (blue) and *w*^*Dah*^*;UAS-Pten-G137E/+;+* (grey) flies. *n* > 55 flies per group. Note that the *repo-GAL4/+* curves in **c**, **d** and **e**, **f** are identical, as these experiments were run in parallel. **g**, **h** qPCR from head RNA shows **g** over-expression of *Pten* in both *w*^*Dah*^*;UAS-Pten/+;repo-GAL4/+* and *w*^*Dah*^*;UAS-Pten-G137E/+;repo-GAL4/+* flies using primers outside of the G137 region and **h** over-expression of wild-type *Pten* in only *w*^*Dah*^*;UAS-Pten/+;repo-GAL4/+* flies using primers designed to amplify the wild-type sequence for G137. *n* = 4 replicates of 10–12 heads per replicate. *p* values are from log-rank tests (**a**–**f**) or Bonferroni’s multiple comparison (**g**, **h**) tests versus control group of that colour

In addition to its lipid phosphatase activity, PTEN exhibits protein phosphatase activity that can act outside of the canonical IIS pathway [30]. To determine whether PTEN’s lipid phosphatase activity was necessary for its effects on lifespan and development, we used a *UAS-Pten-G137E* transgene with a *Drosophila* missense mutation analogous to human *PTEN-G129E*, which impairs PTEN’s lipid phosphatase activity while maintaining its protein phosphatase activity [31]. We first used the inducible GeneSwitch system [32] to confirm that adult-onset ubiquitous over-expression of *Pten* (Fig. 2a, median + 2.3%, *p* = 0.0010), but not *Pten-G137E* (Fig. 2b, median + 0.0%, *p* = 0.54), extended healthy lifespan. We then used *repo-GAL4* to drive expression in glia and found similar effects for glia-specific over-expression of *Pten* (Fig. 2c, median + 2.9%, *p* = 0.0026 versus the nearest control group), but not *Pten-G137E* (Fig. 2d, median + 0.0%, *p* = 0.59 versus the nearest control group), in extending lifespan. In addition, wild-type *Pten*, but not *Pten-G137E*, delayed development when expressed in glia (Fig. 2e, f). We confirmed by qPCR that *UAS-Pten* and *UAS-Pten-G137E* were over-expressed at similar levels and with the expected mutation at the G137 site for *UAS-Pten-G137E* (Fig. 2 g, h), suggesting that differential expression of the transgenes did not underlie their different effects on lifespan and developmental timing. Taken together, these data indicate that PTEN’s lipid phosphatase activity is necessary for its effects on both development and adult lifespan.

### Adult-specific IIS inhibition in glia extends lifespan in a foxo-dependent manner

**Fig. 3:**
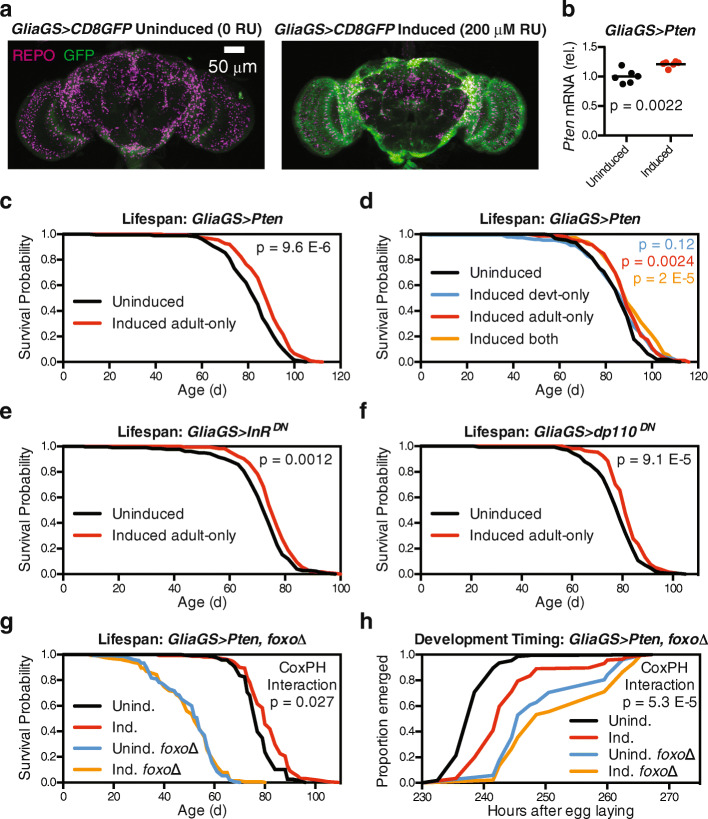
Adult-specific IIS inhibition in glia extends lifespan in a *foxo*-dependent manner. **a** Confocal z-projections show adult (1-week-old) brains with glial nuclei immunostained for REPO (magenta) and glial membranes labelled with mCD8::GFP (green) in 1-week-old *w*^*Dah*^*;+;GSG3285-1/UAS-mCD8::GFP* flies reared on food containing 200 μM RU-486 or vehicle control from 2 days of age. **b** qPCR from head RNA shows over-expression of *Pten* in 2-week-old *w*^*Dah*^*;UAS-Pten/+;GSG3285-1/+* flies reared on food containing 200 μM RU-486 (induced, red) or vehicle control (uninduced, black) from 2 days of age. *n* = 6 replicates of 12 heads per replicate per genotype. **c**–**f** Survival curves show extended lifespan for (**c**, **d**) *w*^*Dah*^*;UAS-Pten/+;GSG3285-1/+*, **e** *w*^*Dah*^*;UAS-InR*^*DN*^*/+;GSG3285-1/+*, and **f** *w*^*Dah*^*;UAS-dp110*^*DN*^*/+;GSG3285-1/+* flies reared on food containing 200 μM RU-486 (red curves) from 2 days of age compared to vehicle control (black curves). However, **d** *w*^*Dah*^*;UAS-Pten/+;GSG3285-1/+* flies reared on food containing 200 μM RU-486 during larval development alone (blue curve) showed no significant change in lifespan, and flies reared on RU-486 throughout both larval development and adulthood (orange curve) showed no additive lifespan extension from adult-only induction. **g** Survival curves show extended lifespan for *w*^*Dah*^*;UAS-Pten/+;GSG3285-1/+* flies (induced, red curve, and uninduced, black curve) and unchanged lifespan for *w*^*Dah*^*;UAS-Pten/+;GSG3285-1,foxo∆/foxo∆* flies (induced, orange curve, and uninduced, blue curve) reared on food containing 200 μM RU-486 from 2 days of age. **h** Egg-to-adult timing shows delayed development for *w*^*Dah*^*;UAS-Pten/+;GSG3285-1/+* larvae (induced, red curve; and uninduced, black curve), and a lesser delay of development for *w*^*Dah*^*;UAS-Pten/+;GSG3285-1,foxo∆/foxo∆* larvae (induced, orange curve; and uninduced, blue curve), reared on food containing 200 μM RU-486. For all lifespans, *n* > 140 deaths counted per group; for development assays, *n* > 40 flies counted per group. *p* values are from unpaired *t* test (**a**), log-rank test versus uninduced (**c**, **e**, **f**), log-rank test for group of that colour versus uninduced (**d**), or Cox proportional hazards (**g**, **h**)

To determine whether effects on development could contribute to later effects on adult longevity, we next used the GeneSwitch system to drive glial *Pten* over-expression with the inducing drug RU-486, using the *GSG3285-1* (*Glia-GS*) driver reported to express specifically in glia [33–35]. We first confirmed by *UAS-mCD8::GFP* expression that *Glia-GS* expressed in glial cells only when flies were fed RU-486 (Fig. 3a). We then over-expressed *Pten* in adult glia starting at 2 days of adult age, which was sufficient to extend lifespan (Fig. 3b, c, median + 5.4%, *p* = 9.6 × 10^−6^). Flies with adult-onset glial *Pten* over-expression showed an overall 37% reduced hazard of death (Fig. 3b, Cox hazard ratio 0.63, 95% confidence interval 0.51–0.78 compared to the uninduced control group). Importantly, the effects on lifespan were reproducible in independent experiments and were not produced by the driver alone in response to RU-486 (Additional file 1: Figure S2a-c). We then tested whether developmental effects of glial *Pten* over-expression could have persistent effects on adult lifespan. We found that glial *Pten* over-expression during development was insufficient to extend lifespan, with no significant interaction between developmental and adult induction (Fig. 3d, Cox proportional hazards interaction *p* = 0.75). These results suggest that *Pten* over-expression in glia during development is dispensable for effects on adult lifespan.

We next investigated other means of IIS inhibition in glia. We found that adult-onset glial expression of either a dominant-negative insulin receptor (*InR*^*DN*^) or a dominant-negative PI3K (*dp110*^*DN*^) significantly extended lifespan (Fig. 3e, f, median + 6.2%, *p* = 0.0012 for *InR*^*DN*^, median + 2.6%, *p* = 9.1 × 10^− 5^ for *dp110*^*DN*^). Notably, whereas ubiquitous reduction of RAS activity downstream of IIS is sufficient to extend lifespan [12], we observed no effect on lifespan for glial expression of dominant-negative RAS (*Ras*^*DN*^, Additional file 1: Figure S2d). Downstream of InR and PI3K, the transcription factor FOXO is necessary for ubiquitous IIS inhibition to extend lifespan, but not for its effects on developmental delay and growth [11]. We thus asked whether FOXO was necessary for the effects we observed in glia. In flies homozygous for the null allele *foxo*^*∆*^, we observed that *Glia-GS* driving *Pten* no longer extended lifespan (Fig. 3g, Cox proportional hazards interaction *p* = 0.027). While the short-lived nature of *foxo*-null flies might place limits on epistasis experiments, previous studies have found that multiple lifespan-extending interventions are able to extend the short lifespan of *foxo*-null flies, including dietary restriction [36, 37], rapamycin treatment [38], constitutive (but not adult-specific) ubiquitous expression of a dominant negative insulin receptor [11], and neurosecretory or gut/fat-body *foxo* over-expression [39]. We therefore concluded that FOXO was likely one necessary downstream mediator through which glial *Pten* over-expression can extend lifespan.

We next investigated whether FOXO was necessary for the effects we observed on development. We observed a blunted effect on egg-to-adult timing in *foxo*-null flies with glial *Pten* over-expression (Fig. 3h, Cox proportional hazards interaction *p* = 5.3 × 10^−5^). Interestingly, however, glial *Pten* over-expression was still able to delay development marginally in *foxo*-null flies (orange versus blue curves in Fig. 3h; *p* = 0.052 by log-rank test), suggesting that FOXO is only partially required for the effects of glial IIS inhibition on development. These data suggest that FOXO is necessary for glial *Pten* over-expression to extend lifespan and is partially required for the effects of glial *Pten* over-expression on development; however, the effects on each life stage can be separated temporally.

### Reduced IIS in astrocyte-like glia extends lifespan without delaying development

**Fig. 4:**
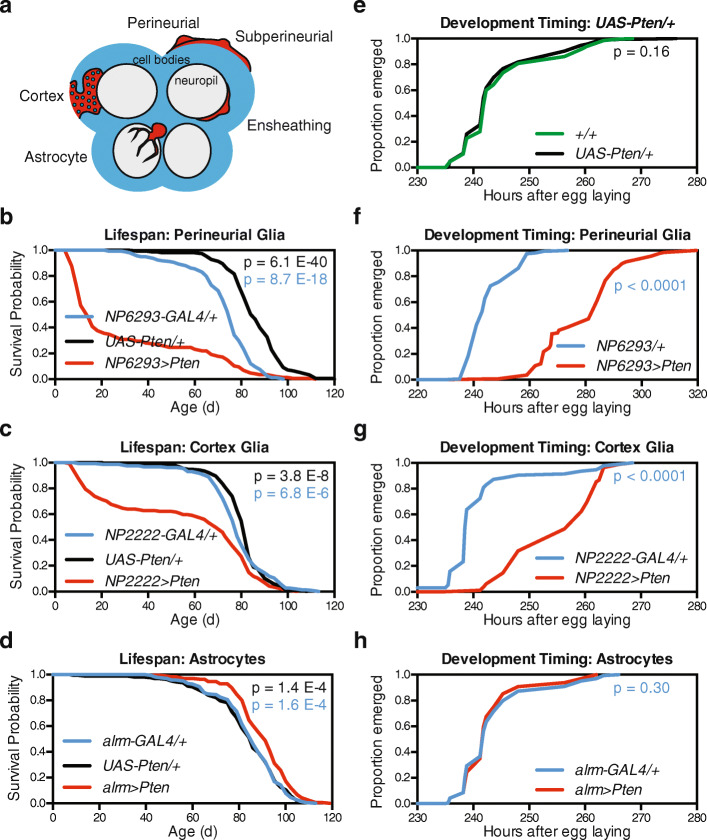
*Pten* over-expression in astrocyte-like glia is sufficient to extend lifespan without delaying development. **a** *Drosophila* glia comprise several distinct subsets, including the perineurial and subperineurial glia that form the blood-brain barrier, cortex glia that surround neuronal cell bodies, ensheathing glia that line the neuropil, and astrocyte-like glia whose processes infiltrate the neuropil. Panel adapted from [40]. **b** Survival curves show reduced lifespan for *w*^*Dah*^*;UAS-Pten/NP6293-GAL4;+* flies (red) compared to control *w*^*Dah*^*;NP6293-GAL4/+;+* (blue) and *w*^*Dah*^*;UAS-Pten/+;+* (black) flies. **c** Survival curves show reduced lifespan for *w*^*Dah*^*;UAS-Pten/NP2222-GAL4;+* flies (red) compared to control *w*^*Dah*^*;NP2222-GAL4/+;+* (blue) and *w*^*Dah*^*;UAS-Pten/+;+* (black) flies. **d** Survival curves show extended lifespan for *w*^*Dah*^*;UAS-Pten/+;alrm-GAL4/+* flies (red) compared to control *w*^*Dah*^*;+;alrm-GAL4/+* (blue) and *w*^*Dah*^*;UAS-Pten/+;+* (black) flies. For all lifespans, *n* > 140 deaths counted per group. **e** Egg-to-adult timing shows unaffected development timing for *w*^*Dah*^*;UAS-Pten/+;+* flies (black) compared to control wDah;+;+ flies (green). **f** Egg-to-adult timing shows delayed development timing for *w*^*Dah*^*;UAS-Pten/NP6293-GAL4;+* flies (red) compared to control *w*^*Dah*^*;NP6293-GAL4/+;+* flies (blue). **g** Egg-to-adult timing shows delayed development timing for *w*^*Dah*^*;UAS-Pten/NP2222-GAL4;+* flies (red) compared to control *w*^*Dah*^*;NP2222-GAL4/+;+* flies (blue). **h** Egg-to-adult timing shows unaffected development timing for *w*^*Dah*^*;UAS-Pten/+;alrm-GAL4/+* flies (red) compared to control *w*^*Dah*^*;+;alrm-GAL4/+* flies (blue). For all development assays, *n* > 70 flies counted per group. *p* values are from log-rank tests versus control group of that colour

We next asked if glia-derived effects on lifespan and development could be separated spatially among individual glial cell subtypes. *Drosophila* central nervous system glia comprise distinct subsets with functions that closely parallel their mammalian counterparts [40]. These subtypes include the perineurial and subperineurial glia that form the *Drosophila* blood-brain barrier, cortex glia that surround neuronal cell bodies, ensheathing glia that line the neuropil, and astrocyte-like glia whose processes infiltrate the neuropil (Fig. 4a). We therefore tested if *Pten* over-expression using GAL4 lines for these individual glia subtypes [41, 42] would recapitulate the effects of pan-glial expression on lifespan and development. We found that *Pten* dramatically shortened lifespan when over-expressed in either perineurial glia (*NP6293-GAL4*) or cortex glia (*NP2222-GAL4*) (Fig. 4b, c) but had no clear effect on lifespan in subperineurial (*NP2276-GAL4*) or ensheathing (*mz0709-GAL4*) glia (Additional file 1: Figure S3a-b). In contrast, *Pten* over-expression in astrocyte-like glia (*alrm-GAL4*) alone was sufficient to extend lifespan (Fig. 4d, median + 5.4%, *p* = 1.6 × 10^−4^ versus the nearest control group). Flies with astrocyte-specific *Pten* over-expression showed an overall 34% reduced hazard of death (Cox hazard ratio 0.66, 95% confidence interval 0.53–0.83 compared to nearest control group). This lifespan extension was reproducible in independent experiments (Additional file 1: Figure S4a) and for flies expressing *UAS-InR*^*DN*^ in astrocyte-like glia (Additional file 1: Figure S4b). Interestingly, we observed the inverse pattern for the effects of each glial subtype on developmental timing: *Pten* over-expression strongly delayed development when restricted to either perineurial or cortex glia (Fig. 4e–g), moderately delayed development in subperineurial or ensheathing glia (Additional file 1: Figure S3c-d), and had no effect on development in astrocyte-like glia (Fig. 4h). The detrimental effects of IIS inhibition in perineurial and cortex glia are in line with previous findings that these two glial subtypes require IIS for their proliferation during larval development [43]. However, an important caveat is that GAL4 drivers can be active in multiple tissues or cell types. To address this, we dissected larvae expressing mCD8::GFP under the control of each GAL4 line. We observed GFP in central nervous system cells corresponding to each glial subtype; however, we also observed GFP in the salivary gland and/or fat body for *NP2222-GAL4*, *mz0709-GAL4*, and *NP2276-GAL4* (Additional file 1: Table S1). In contrast, we observed high specificity of GFP expression for *Glia-GS* and *alrm-GAL4*, suggesting that the increases in lifespan we observed could be attributed to glia generally and astrocyte-like glia specifically.

## Discussion

We have described here that IIS inhibition in glia, and in astrocyte-like glia specifically, can extend *Drosophila* lifespan. This work adds glial cells to a list of tissues in which IIS inhibition can extend lifespan, including skeletal muscle [18], fat body [13, 14], and gut [19]. Notably, glial cells comprise roughly 10% of the total cells in the adult *Drosophila* nervous system, with astrocyte-like glia representing roughly 3.4% of total nervous system cells [44]. Our results showing that this small cell population can modulate lifespan underscore their crucial role in maintaining not only nervous system function but also organism-wide health.

One caveat to consider in this study is that the extensions in lifespan we observe are relatively small (2 to 6% increase in median lifespan with overall 34–37% reduction in the hazard of death). However, our results are highly significant and reproducible and fall in the range we observe for ubiquitous over-expression of PTEN (Fig. 2, 2.3% increase). Moreover, glial-specific PTEN over-expression does not result in impaired organismal size or fecundity (Fig. 1) as observed for some models of ubiquitous IIS inhibition [17]. Our results suggest that IIS inhibition restricted to glia can produce a large proportion of the lifespan extension seen with ubiquitous IIS inhibition, while avoiding some of the trade-offs with organismal size and reproduction. What then explains the small magnitude of our increases in median lifespan? One possibility is that relative increases in lifespan are diminished in populations with very healthy long-lived control flies (medians of > 80 days in many experiments for this study). This possibility is supported by studies on one of the most well-characterised long-lived *Drosophila* mutants, the insulin receptor substrate orthologue *chico.* Flies heterozygous for *chico* show up to 36% increase in median lifespan when their wild-type siblings have a median lifespan of 40–45 days [7], but only 12% increase in median lifespan when their wild-type siblings have a median lifespan of 65–70 days [12]. When healthily long-lived controls have even higher median lifespans of > 80 days, even ubiquitous interventions such as inhibition of the RAS/ERK pathway extend lifespan by 4 to 6% [12]. However, there remains a real possibility that modulating one branch of a pathway in one cell type may not be able to extend lifespan to the extent seen with systemic interventions like ubiquitous genetic changes, dietary restriction, or pharmacological treatments. The relatively small magnitudes of lifespan extension in our studies on astrocytes therefore suggest that future studies will benefit from investigating functional consequences of IIS inhibition in astrocytes and, crucially, whether astrocytes modulate healthy lifespan and function with age in more complex species.

Several recent studies lend support to the hypothesis that glia, and astrocytes specifically, play key roles in nervous system ageing across species. Transcriptomic analysis of human brain tissue shows that many genes specific to astrocytes and oligodendrocytes, but few genes specific to neurons, change their regional expression patterns with age [26]. Two other studies using cell-type-specific RNA-seq analysis of ageing mouse brain have also found strong ageing transcriptional signatures in astrocytes, including upregulation of genes involved in synapse elimination [45, 46]. In *Drosophila*, an extensive analysis of single-cell transcriptomes from ageing fly brains has found that glia, but not neurons, show a clear ageing trajectory of transcriptional patterns [25], with additional analysis revealing a particularly strong age-related pattern for astrocyte-like glia [27]. Taken together, these transcriptomic studies suggest that astrocytes may be one of the cell types that play the greatest role in the nervous system during ageing across animal species. Importantly, this has been corroborated recently by landmark findings in *C. elegans* showing that a small population of astrocyte-like glia can modulate lifespan via secretion of one or more yet-unidentified neuropeptides [47]. Taken together, these studies and our results highlight astrocyte-like glia as an evolutionarily conserved locus that can extend healthy lifespan among multiple invertebrate species. Future studies to identify the exact mechanisms by which astrocytes modulate organism-wide ageing could therefore have applications that extend to more complex animals including humans.

Mammalian astrocytes and *Drosophila* astrocyte-like glia share important functions that could contribute to their ability to modulate nervous system ageing or lifespan. In both mammals and *Drosophila*, astrocytes express transporters that clear the neurotransmitters glutamate [48] and GABA [49] from synapses; loss of either transporter in glia results in locomotor dysfunction and neurodegeneration [49, 50]. In addition, mammalian astrocytes actively eliminate synapses during postnatal development through pathways that require the engulfment receptor MEGF10 [51]. Similarly, *Drosophila* astrocyte-like glia require the *Megf10* orthologue *Draper* to clear neuronal debris during metamorphosis [52, 53]. It is worth noting, however, that *Drosophila* cortex glia [54] and ensheathing glia [42] also play important roles in debris clearance during development and injury, so additional glial subtypes may be involved in debris clearance depending on the context and age of the organism. In the ageing mouse brain, astrocytes show increased expression of a number of genes involved in synapse elimination and inflammatory responses, potentially contributing to neuronal damage with age [45, 46]. Indeed, recent transcriptomic studies from either single-nuclei or sorted astrocyte populations have shown that reactive inflammatory astrocytes accumulate in the aged human brain and in the brains of Alzheimer’s disease patients [55, 56]. The diversity of the beneficial and toxic functions of astrocytes thus present a number of directions for future research on astrocyte function both in disease and in healthy ageing.

Finally, our results pose an important question for understanding the evolution of IIS regulation among different cell types. If astrocytic IIS is lifespan-limiting and does not appear to play a central role in development, why has evolution not favoured a dampening of this pathway’s activity in astrocytes? Some hints to answering this question may come from the beneficial role played by glial IIS in clearing debris in the nervous system. For instance, signalling through the insulin receptor and AKT upregulates expression of *Draper* in *Drosophila* glia after neuronal injury [57]; conversely, decreased IIS in *Drosophila* glia reduces their ability to clear axonal debris after axotomy [58]. When taken together with our results, these studies present a compelling case for wound healing as one beneficial component of the antagonistically pleiotropic effects of IIS in astrocytes. The functions of IIS in wound healing also extend beyond the nervous system, as IIS signalling through both FOXO inhibition and TOR activation is required for proper epidermal wound healing responses [59]. Therefore, although sustained IIS in some cell types may not have been selected for its role in development, its role in wound healing may underlie this example of cell-type-specific antagonistic pleiotropy. One challenge of ageing research is now to identify whether IIS inhibition in specific downstream pathways or in specific cell subtypes can promote healthy ageing while leaving intact beneficial functions such as wound healing.

## Conclusions

Our results identify glia, and astrocyte-like glia specifically, as cell types through which IIS inhibition can extend lifespan in *Drosophila*. We have further shown that the trade-off between extended lifespan and delayed development can be compartmentalised among glial cell subtypes, with IIS in astrocyte-like glia as a major modulator of lifespan but not development. As whole-organism IIS inhibition extends lifespan in a broad array of organisms including *Drosophila* and mice [4, 5], it will be of great interest to determine whether astrocytes play a role in ageing in more complex species. Finally, our results add weight to the emerging paradigm that age-related changes in astrocytes could underlie the susceptibility of aged brains to the increasingly prevalent and costly neurodegenerative diseases of ageing, supporting the investigation of astrocyte-targeted interventions to address these devastating diseases.

## Methods

### Fly stocks and husbandry

Stocks were maintained and experiments conducted at 25 °C on a 12 h:12 h light to dark cycle at 60% humidity, on food containing 10% (w/v) brewer’s yeast, 5% (w/v) sucrose, and 1.5% (w/v) agar. For experiments involving the GeneSwitch system, RU-486 (Sigma) dissolved in ethanol was added to the food at a final concentration of 200 μM, with 2 mL/L ethanol used as the vehicle control condition.

The wild-type stock Dahomey was collected in 1970 in Dahomey (now Benin) and has since been maintained in large population cages with overlapping generations on a 12 h:12 h light to dark cycle at 25 °C. The *white Dahomey* (*w*^*Dah*^) stock was derived by incorporation of the *w*^*1118*^ mutation into the outbred Dahomey background by backcrossing. All fly stocks in this study were back-crossed for six or more generations into the outbred *w*^*Dah*^ background, except for experiments in Additional file 1: Figure S1b where stocks were back-crossed for six generations into an inbred *w*^*1118*^ background. Fly stocks used were *repo-GAL4* (Bloomington #7415); *Actin5c-GeneSwitch* [60]; *GSG3285-1* (*Glia-GeneSwitch*) [33, 35]; *NP6293-GAL4* (Kyoto #105188); *NP2222-GAL4* (Kyoto #112830); *NP2276-GAL4* (Kyoto #112853); *mz0709-GAL4* (3rd chromosome) [42]; *alrm-GAL4-#2* (3rd chromosome) [42]; *UAS-Pten* [28]; *UAS-Pten3-G137E* [31]; *UAS-InR*^*DN*^ (*InR*^*K1409A*^, Bloomington #8252); *UAS-dp110*^*DN*^ (*dp110*^*D954A*^ Bloomington #25918); *UAS-Ras*^*DN*^ (*Ras*^*N17*^ [61]); *UAS-mCD8::GFP* (Bloomington #5130); and *foxo*^*∆*^ [11].

### Survival analysis

Lifespan assays were carried out as described in detail in [62], with female flies used for all lifespan experiments. From the eggs collected for each set of parental crosses, the progeny that emerged as adults within a 24-h window were collected and allowed to mate for 48 h, after which they were separated into single-sex vials containing either standard food (GAL4 experiments) or vehicle- or RU-486-containing food (GeneSwitch experiments) at a density of 12 or 15 individuals per vial. Flies were randomly allocated to treatments with sample sizes of > 100 flies per condition as recommended in [62]. Flies were transferred to fresh vials three times a week, with deaths and censors scored during each transferral. Microsoft Excel was used to calculate and plot survival proportions as described in [62]. Where possible, experimenters were blinded to the genotypes of flies in each lifespan experiment.

### Egg-to-adult timing

Flies from parental crosses were allowed to lay eggs on grape juice plates (recipe and details available in [62]) for 3-h time windows throughout the light cycle. Twenty-four hours later, first-instar larvae were collected and transferred to vials containing either standard food (GAL4 experiments) or vehicle- or RU-486-containing food (GeneSwitch experiments) at a density of 50 larvae per vial. Starting 9 days after egg laying, the number of female adult flies emerging from each vial was counted every 2 to 4 h during the light cycle until all adults had emerged.

### Fly weight measurements

Flies were collected for lifespan assays and allowed to age to 1 week of age. The wet weights of individual cold-anaesthetised female flies were assessed using a Mettler Toledo AT201 balance with a sensitivity of 0.01 mg.

### Fecundity measurements

In parallel to lifespan experiments, female flies were collected for quantifying the number of eggs laid. Twelve flies per vial were allowed to lay eggs on vials containing standard food for 24 h at the ages indicated, after which the vials were frozen to prevent larval hatching. After all vials were collected, a Logitech HD Pro C920 camera was used to capture images of the food surface for each vial. These images were processed for automated egg counting using QuantiFly software described in [63]. The software was trained using a subset of vials from each experiment in which eggs were counted by hand.

### Quantitative real-time PCR (qPCR)

Total RNA was isolated from 10 to 12 adult fly heads per replicate using standard Trizol (Invitrogen) protocols. RNA samples were treated with Turbo DNAse (Invitrogen) and converted to cDNA using oligod(T) primers and Superscript II reverse transcriptase (Invitrogen). Quantitative RT-PCR was performed using Power SYBR Green PCR Master Mix (ABI) in the Quant Studio 6 Flex system. Relative quantities of transcripts were determined using the relative standard curve method normalised to *Tubulin84B*. *Pten* primers were designed to amplify both wild-type and *G137E* mutant *UAS-Pten* transgenes (*Pten_tot* primers) or to amplify only wild-type but not *G137E* mutant *UAS-Pten* transgenes (*Pten_wt* primers). Primer sequences were as follows: *Pten_tot_for*: AATTTCGCGGGAGAGTAGCTG; *Pten_tot_rev*: ACGGCTACAACATTGGACGA; *Pten_wt_for*: GTCCAATGTTGTAGCCGTGC; *Pten_wt_rev*: AGATCATGGTACCGGTTCTGC; *Tub84B_for*: TGGGCCCGTCTGGACCACAA; *Tub84B_rev*: TCGCCGTCACCGGAGTCCAT.

### Immunofluorescence and image analysis

Immunofluorescence in adult brains was carried out as described in [64]. Briefly, adult brains were dissected in PBT (phosphate-buffered saline (PBS) containing 0.3% Triton X-100) and fixed in 4% paraformaldehyde in PBT for 20 min at room temperature. After fixation, brains were washed with PBT three times for 20 min at room temperature, then blocked in PBT containing 10% BSA (PBT-BSA) for 1–2 h at room temperature. For primary antibody treatment, samples were incubated in PBT-BSA containing the primary antibody overnight at 4 °C. The following primary antibodies were used: mouse anti-REPO (Developmental Studies Hybridoma Bank #8D12-s, 1:100) and rabbit anti-GFP AlexaFluor 488 (Invitrogen A21311, 1:250). After primary antibody incubation, brains were washed with PBT three times for 20 min at room temperature, then incubated in PBT-BSA containing the secondary antibody for 2 h at room temperature. The following secondary antibodies were used: goat anti-mouse AlexaFluor 568 (Invitrogen A11019, 1:250) and donkey anti-mouse AlexaFluor 488 (Invitrogen A21202, 1:250). Brains were then washed four times with PBT for 20 min at room temperature and mounted on slides in Vectashield containing DAPI (Vector Laboratories).

Image stacks were obtained on a Zeiss LSM700 confocal microscope using a × 10 or × 20 objective for whole-brain imaging and a × 63 objective for imaging of glial cell layers in the optic lobe. For whole brains, stacks of 10-μm Z-distance and 11 images per stack were taken. Images were quantified using Fiji software [65]. For whole brains, confocal stacks were merged into a single plane by using the maximum projection function. The central brain was then traced by hand to measure the area of the maximal cross-section. For images of optic lobe glial layers, stacks of 1.0-μm Z-distance and 13 images per stack were taken. Confocal stacks were merged into a single plane by using the maximum projection function, after which the number of REPO-positive nuclei were counted by hand to estimate the density of glial nuclei in this region. To determine the extent of glial membranes, a threshold was set for GFP, and the area above threshold within this region was measured. This value was then divided by the number of glial nuclei in the image to determine the mean GFP-positive area per glial nucleus.

### Statistical analysis

The statistical test used for each experiment is indicated in the figure legend. Log-rank tests on lifespan and developmental timing data were performed in Microsoft Excel [62] or Graphpad Prism 6.0a. ANOVA analyses on fly weight, fecundity, immunofluorescence, and qPCR data were performed in Graphpad, with data displayed as mean with all data points shown. Cox proportional hazards tests were performed in R using the ‘survival’ package (Terry Therneau). For all statistical tests, *p* < 0.05 was considered significant.

## Supplementary information

**Additional file 1.** 4 supplementary figures, 1 supplementary table.

**Additional file 2.** Raw data for lifespan experiments.

## Data Availability

Raw data for lifespan experiments are available in Additional file 2. Other datasets used in the current study are available from the corresponding author on reasonable request.
