# Supplementary material for: Independent glial subtypes delay development and extend healthy lifespan upon reduced insulin-PI3K signalling
Source: BMC Biol. 2020 Sep 14;18:124. doi: 10.1186/s12915-020-00854-9 (PMC7490873; doi:10.1186/s12915-020-00854-9)
Supplement: Supplementary file 1 — Additional file 1. 4 supplementary figures, 1 supplementary table. [file 12915_2020_854_MOESM1_ESM.pdf]

Supporting Figures and Table for:

Independent glial subtypes delay development and extend healthy lifespan upon reduced insulin-PI3K signalling

Nathaniel S. Woodling[1], Arjunan Rajasingam[1], Lucy J. Minkley[1], Alberto Rizzo[1], and Linda Partridge[1,2]

[1] Institute of Healthy Ageing and Department of Genetics, Evolution and Environment, University College London, Darwin Building, Gower Street, London WC1E 6BT, United Kingdom

[2] Max Planck Institute for Biology of Ageing, Joseph-Stelzmann-Strasse 9b, 50931 Cologne, Germany

Correspondence should be addressed to:
Linda Partridge
Institute of Healthy Ageing
University College London
l.partridge@ucl.ac.uk

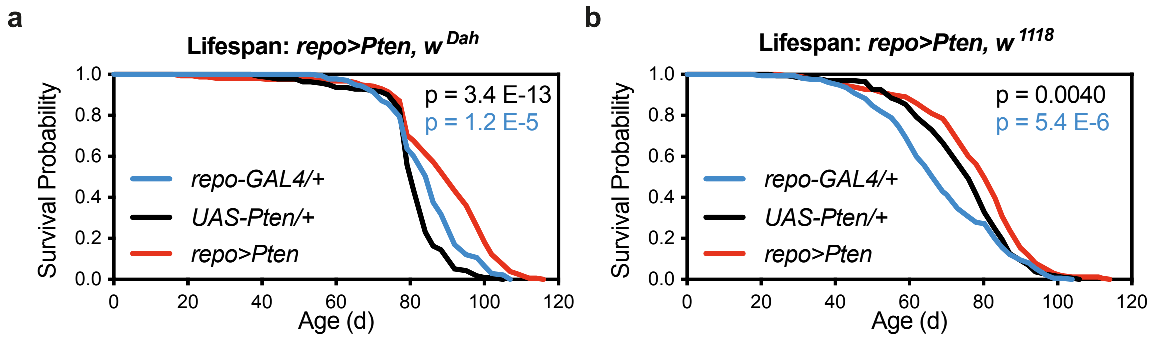

Figure S1. Over-expression of *Pten* in glia reproducibly extends lifespan in multiple genetic backgrounds.

(**a**) Survival curves show extended lifespan for w^{Dah}*;UAS-Pten/+;repo-GAL4/+* flies (red) compared to control w^{Dah}*;+;repo-GAL4/+* (blue) and w^{Dah}*;UAS-Pten/+;+* (black) flies.
(**b**) Survival curves show extended lifespan for w^{1118}*;UAS-Pten/+;repo-GAL4/+* flies (red) compared to control w^{1118}*;+;repo-GAL4/+* (blue) and w^{1118}*;UAS-Pten/+;+* (black) flies.
For all lifespans, n>130 deaths counted per group; p-values are from log-rank test versus control group of that colour.

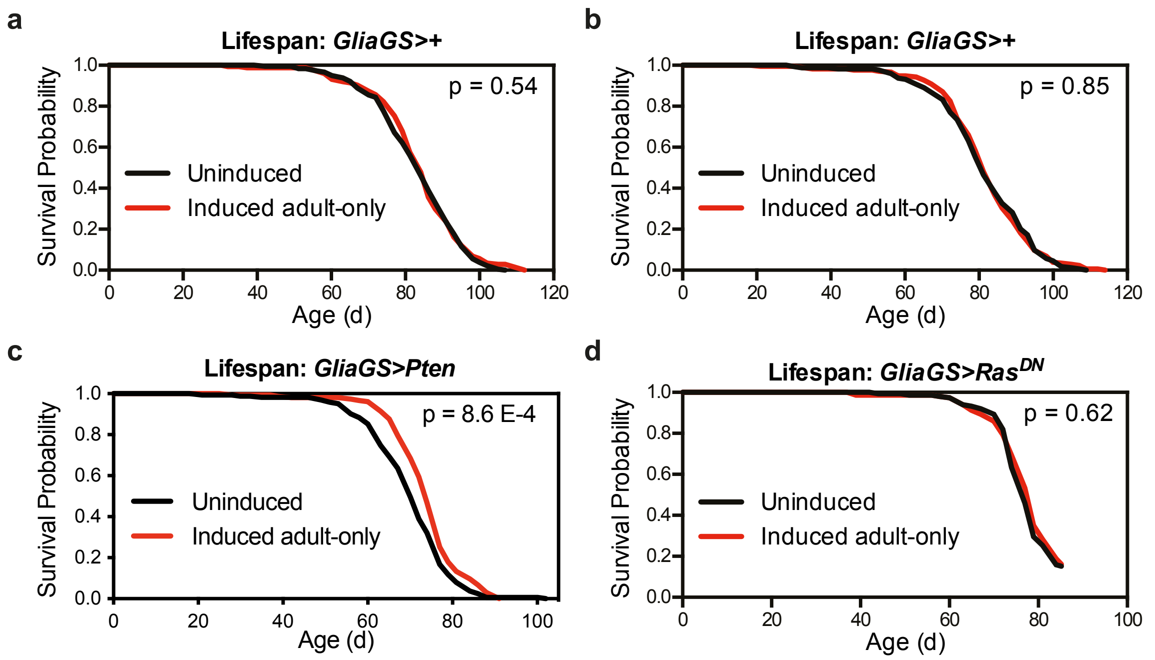

Figure S2. Effects of driver-alone controls and glial over-expression of *Pten* or *Ras*^{DN} during adulthood.

(**a-b**) Survival curves show no significant change in lifespan for driver-alone *w^{Dah};+;GSG3285-1/+* flies reared on food containing 200µM RU-486 (induced, red) from 2 days of age compared to sibling flies on vehicle control food (uninduced, black).

(**a**) Trial 1 was run in parallel to the experiment shown in main text **Figure 3c**.

(**b**) Trial 2 was run in parallel to the experiments shown in main text **Figure 3e-f**.

(**c**) Survival curves show extended lifespan for *w^{Dah};UAS-Pten/+;GSG3285-1/+* flies reared on food containing 200µM RU-486 (induced, red) from 2 days of age compared to sibling flies on vehicle control food (uninduced, black).

(**d**) Survival curves show no significant change in lifespan for *w^{Dah},UAS-Ras^{DN}/w^{Dah};+; GSG3285-1/+* flies reared on food containing 200µM RU-486 (induced, red) from 2 days of age compared to sibling flies on vehicle control food (uninduced, black).

For all lifespans, n>120 deaths counted per group; p-values are from log-rank test versus uninduced control.

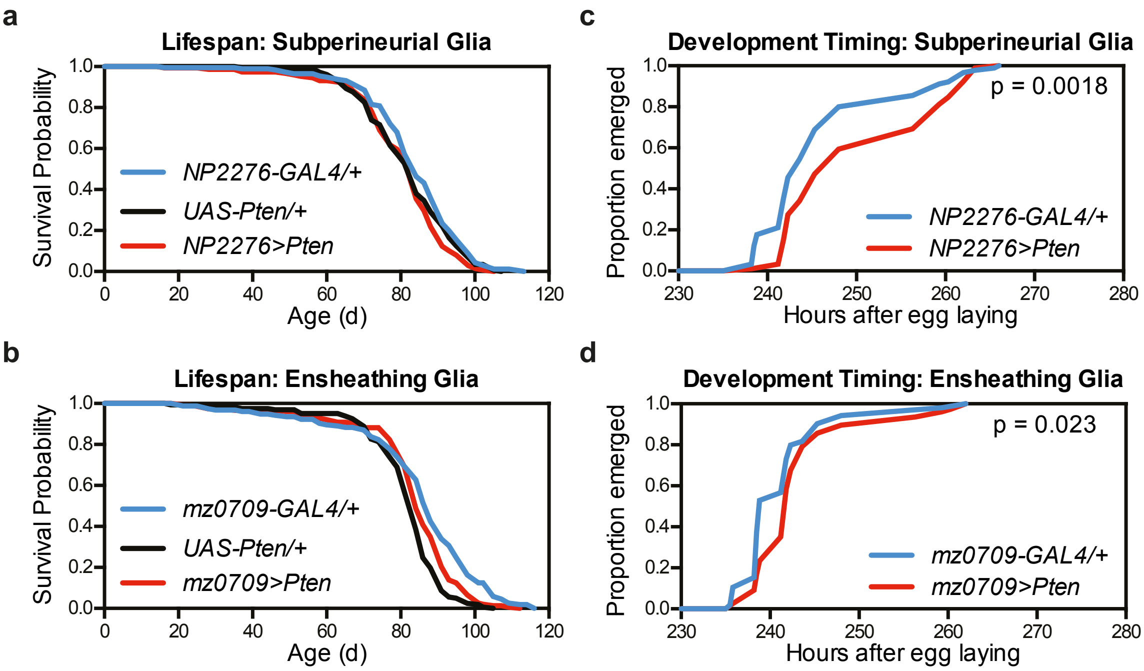

Figure S3. Over-expression of *Pten* in subperineurial or ensheathing glia does not extend lifespan but delays development.

(**a**) Survival curves show no increase in lifespan for w^{Dah}*;UAS-Pten/NP2276-GAL4;+* flies (red) compared to control w^{Dah}*;NP2276-GAL4/+;+* (blue) and w^{Dah}*;UAS-Pten/+;+* (black) flies.

(**b**) Survival curves show no increase in lifespan for w^{Dah}*;UAS-Pten/+;mz0709-GAL4/+* flies (red) compared to control w^{Dah}*;+;mz0709-GAL4/+* (blue) and w^{Dah}*;UAS-Pten/+;+* (black) flies.

(**c**) Egg-to-adult timing shows delayed developmental timing in w^{Dah}*;UAS-Pten/NP2276-GAL4;+* flies (red) compared to control w^{Dah}*;NP2276-GAL4/+;+* (blue) flies.

(**d**) Egg-to-adult timing shows delayed developmental timing in w^{Dah}*;UAS-Pten/+;mz0709-GAL4/+* flies (red) compared to control w^{Dah}*;+;mz0709-GAL4/+* (blue) flies.

For all lifespans, n>140 deaths counted per group. For developmental timing, n>70 flies counted per group. p-values are from log-rank tests versus driver control group.

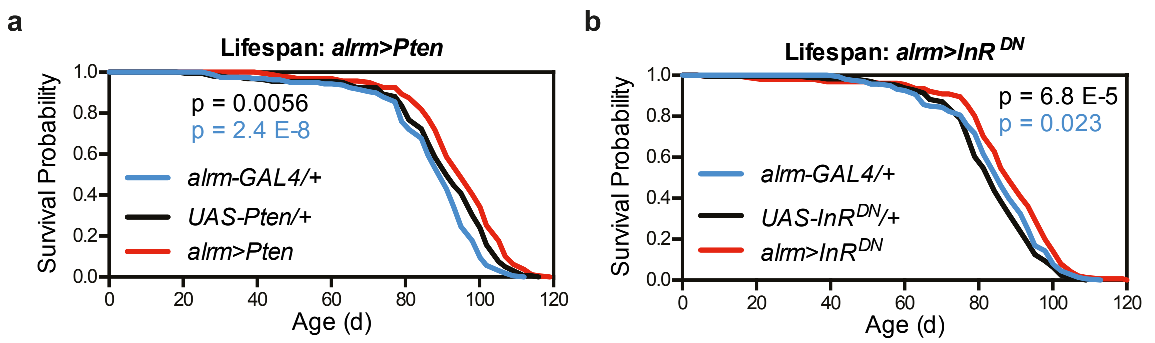

Figure S4. Over-expression of *Pten* or *InR*^{DN} in astrocyte-like glia extends lifespan. (**a**) Survival curves show extended lifespan for *w*^{Dah}*;UAS-Pten/+;alrm-GAL4/+* flies (red) compared to control *w*^{Dah}*;+;alrm-GAL4/+* (blue) and *w*^{Dah}*;UAS-Pten/+;+* (black) flies. (**b**) Survival curves show extended lifespan for *w*^{Dah}*;UAS- InR*^{DN} */+;alrm-GAL4/+* flies (red) compared to control *w*^{Dah}*;+;alrm-GAL4/+* (blue) and *w*^{Dah}*;UAS- InR*^{DN} */+;+* (black) flies. For all lifespans, n>140 deaths counted per group. p-values are from log-rank tests versus control group of that colour. Note that the *alrm-GAL4/+* curve in (**b**) is identical to the *alrm-GAL4/+* curve in main text **Figure 4d**, as these experiments were run in parallel.

Driver	Reported Expression	n larvae	CNS expression pattern	Salivary Gland	Imaginal Discs	Fat Body
No Driver	none	3	-	-	-	-
GSG3285-1 +RU 200μM	pan-glial	5	moderate in cells throughout CNS	-	-	-
repo-GAL4	pan-glial	5	strong in cells throughout CNS	weak	-	-
NP2222-GAL4	cortex glia	5	strong in cell body layers of CNS	strong	-	-
NP2276-GAL4	subperineurial glia	4	moderate in surface cells of CNS	-	-	moderate
mz0709-GAL4	ensheathing glia	4	weak in non-surface CNS cells	strong	-	-
alrm-GAL4	astrocyte glia	5	strong in non-surface CNS cells	-	-	-

Table S1. Expression pattern of GAL4 and GeneSwitch driver lines used for developmental timing and lifespan studies. Flies for each driver line were crossed to flies with the *UAS-mCD8::GFP* transgene. L3 larvae from these crosses were dissected and examined under fluorescence microscopy to assess GFP expression levels in the central nervous system (CNS), salivary glands, imaginal discs, and fat body. “-” indicates no observable GFP fluorescence for the indicated tissue in any larvae.
